# Some Lessons Learned on the Impact of the Storage Conditions, Syringe Wash Solvent, and the Way of GC-MS Injection on the Reproducibility of Metabolomic Studies

**DOI:** 10.3390/metabo13010075

**Published:** 2023-01-03

**Authors:** Ilya Kurbatov, Olga Kiseleva, Viktoriia Arzumanian, Georgii Dolgalev, Ekaterina Poverennaya

**Affiliations:** Institute of Biomedical Chemistry, 119121 Moscow, Russia

**Keywords:** two-dimensional gas chromatography–mass spectrometry, metabolomics, blood plasma, reproducibility, storage, injection

## Abstract

Metabolomics based on two-dimensional gas chromatography coupled with mass spectrometry is making high demands on accuracy at all stages of sample preparation, up to the storage and injection into the analytical system. In high sample flow conditions, good repeatability in peak areas and a list of detectable metabolites is sometimes challenging to obtain. In this research, we successfully obtained good repeatability for the peak areas of MSFTA-derivatives of 29 core blood plasma metabolites. Six different strategies of storage and injection were investigated and evaluated for the reproducibility of the obtained data. As the essential factors, we considered popular GC-MS syringe washing solvents (methanol and pyridine); storage conditions (freshly prepared samples and stored for 24 h in ambient temperature or in the refrigerator); scheme of injection (one injection per intact vial or three sequential injections per vial). Our GC×GC-MS results demonstrated that the usage of pyridine as a syringe wash solvent and triple injecting the sample from the same vial was the most appropriate for minimizing the coefficient of variation (CV) of the results obtained (in general, <10%). The prolonged storage of samples does not have a noticeable effect on the change in the areas of chromatographic peaks of metabolites, although it reduces CV in some cases. These storage and injection recommendations can be used in future study protocols for the GC×GC-MS analysis of blood plasma.

## 1. Introduction

Silylation is one of the most versatile derivatization procedures enhancing the performance of the analysis of small molecules via gas chromatography coupled with mass spectrometry (GC-MS). Silylation results in substituting active hydrogen of -OH, NH2, -SH, and other polar functional groups with a non-polar alkylsilyl (most frequently trimethylsilyl) radical. Hence, more volatile, less polar, and thermally stable silylated derivatives are routinely obtained using a broad spectra of various reagents [1,2]. Numerous compounds have been synthesized to be effectively used in silylation selection based on their reactivity toward the target compounds, the stability of the derivative, and the effect of the by-products on the analytical system. N-Trimethylsilyl-N-methyl trifluoroacetamide (MSTFA) possessing optimal silyl donor ability is one of the reagents of choice [3]. Another significant advantage of MSTFA is that it is unnecessary to be removed from the sample prior to GC-MS analysis. However, several principal metabolites may produce relatively unstable trimethylsylil(TMS)-derivatives, requiring them to be sealed, properly stored, and analyzed to prevent decay and safe analytical value [4].

In this technical note, we explored the intensity variability of m/z features of MSTFA-derived small molecules in blood plasma. As the essential factors that potentially affect the reproducibility of chromato–mass spectrometric experiments, we considered a popular GC-MS syringe washing solvent (MeOH and pyridine); storage conditions (freshly prepared samples and stored 24 h in ambient temperature or in the refrigerator); and the scheme of injection (one injection per intact vial or three sequential injections per vial). When choosing a solvent for flushing the injection system, one should consider the reactivity of both the solvent itself and the derivatives. By analogy with water, wash solvents popular in gas chromatography (for example, methanol) can react with a silylation agent [5] and destabilize TMS derivatives of target metabolites. Another variable experimental parameter is the conditions and duration of the storage of samples prepared for analysis, because violation of these storage conditions may contribute to misleading results. To minimize blood plasma metabolite changes, it is important to use proper storage conditions of derivatized metabolites to ensure the fidelity of results.

Interest in the effect of vial integrity on the reproducibility of results is due to the high sensitivity of TMS-derived metabolites to moisture [6], which can potentially enter the vial from the air. Based on these considerations, to assess the reproducibility of GC×GC-MS analysis, we tested six combinations of experimental conditions for the metabolomic analysis of blood plasma. Our results demonstrated that pyridine as a syringe wash solvent and three injections from the same vial as an injection strategy was the most appropriate combination for minimizing the coefficient of variation (CV) of the results obtained. At the same time, the storage of samples does not have a noticeable effect on the change in the areas of chromatographic peaks of metabolites, although it reduces CV in some cases. These storage and injection recommendations can be used in future study protocols for the GC×GC-MS analysis of blood plasma.

## 2. Materials and Methods

### 2.1. Design of the Experiment

**Figure 1:**
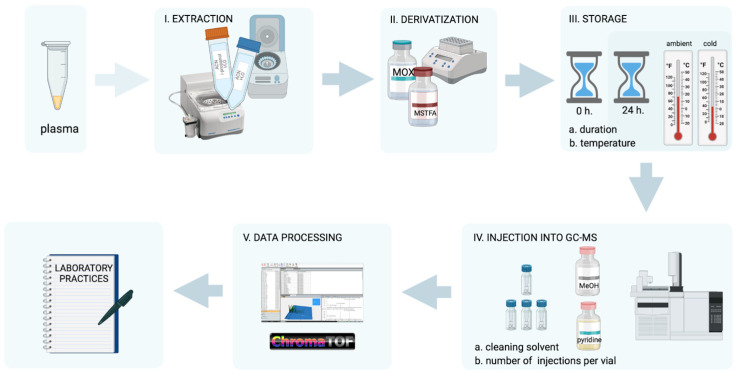
Scheme of the experiment. Blood plasma samples were uniformly prepared: we extracted low molecular weight compounds, then dried (**I**), methoxylated, and derivatized (**II**) the extracts. Our experiment implied three options for sample storage conditions before injection into the GCxGC-MS system (**III**): immediate loading or storage for 24 h (at ambient temperature and in a refrigerator at +4 °C). We washed the injection system with pyridine or methanol to evaluate the effect of the solvent used for syringe wash on the final signal on the chromatogram (**IV**) between injections. At the same stage (**IV**), we tested two schemes of sample loading into the chromatograph: we injected the sample from the same vial three times or used three intact vials. The resulting GCxGC-MS data were processed in a unified manner using the LECO ChromaTOF (**V**) software (v. 5.51) and statistically processed to develop final recommendations for sample storage and injection.

We uniformly prepared plasma samples (Figure 1I–III) from a healthy donor to assess how the intensities of GCxGC-MS signals from human plasma core metabolites change. We analyzed the influence of several factors: duration of storage (sample injection immediately after derivatization or after 24 h) and temperature (samples stored for 24 h after derivatization at room temperature and +4 °C) of prepared samples, as well as features of injection into the chromato–mass spectrometric system (using pyridine or methanol for the pre-injection and post-injection solvent wash, as well as the intactness of the vial (Figure 1IV) containing the sample).

Thus, we processed 36 files of GCxGC-MS spectra of blood plasma samples: for each of the wash solvents, we considered three options for storage conditions (injection immediately after completion of sample preparation, within 24 h of the finish of sample preparation—at room temperature, and within 24 h of the finish of sample preparation—at +4 °C) and two types of injection (every injection from the intact vial or triple injection from the same vial). For each combination of laboratory conditions, we obtained three technical repetitions of the GCxGC-MS experiment.

The resulting chromatograms were processed statistically and interpreted (Figure 1V). The general scheme of the experiment is presented in Figure 1, and the details of each experimental block are discussed separately in the following sections.

### 2.2. Chemicals

The derivatization reagents N-methyl-N-(trimethylsilyl)trifluoroacetamide (MSTFA), methoxyamine hydrochloride (MOX), pyridine, and commercially available mix of fatty acid methyl esters (Supelco 37 Component FAME Mix) used to calculate retention indices were all obtained from Sigma-Aldrich (St. Louis, MO, USA). Methanol (HPLC-MS grade) was purchased from J.T. Baker (Phillipsburg, NJ, USA).

### 2.3. Sample Preparation

A detailed description of the sample preparation procedure used for the present study can be found elsewhere [7]. All aliquots were obtained with Research plus single-channel pipettes (Eppendorf, Germany) calibrated single-channel pipettes of 20, 100, and 1000 µL. Blood plasma was stored at −80 °C. For the extraction of metabolites, mixtures of acetonitrile/isopropanol/water (3:3:2, *v*./*v*./*v*., Solvent 1) and acetonitrile/water (1:1, *v*./*v*., Solvent 2) were used. Before extraction, solvents were degassed in a stream of nitrogen to remove residual oxygen that can oxidize some metabolites (thiols or antioxidants). After degassing, Solvent 1 was stored at −20 °C, and Solvent 2 was stored at +4 °C.

An aliquot (30 µL) of homogenized blood plasma was diluted in Solvent 1 (500 µL), vortexed and shaken for 5 min at 4 °C, after that centrifuged for 2 min at 14,000× *g*, room temperature. Then supernatant (450 µL) was vacuum-dried in SpeedVac evaporator at 40 °C (Eppendorf, Hamburg, Germany). The cycle of extraction and drying was repeated with Solvent 2 (450 µL). Dried extracts were oxidized with 10 µL of freshly prepared methoxyamine hydrochloride (20 mg/mL in pyridine) at 30 °C for 90 min on a thermoshaker (ThermoMixer C, Eppendorf, Hamburg, Germany) at 1300 rpm. Next, the samples were derivatized by 91 µL of MSTFA at 37 °C for 30 min at a thermoshaker at 1300 rpm. The final reagent volume included a mixture of the retention time standards (FAMEs). After extraction and derivatization, samples were stored for 24 h or submitted for GC×GC-MS acquisition immediately, according to experimental design.

### 2.4. GC×GC-MS Analysis

GC×GC-MS analysis was performed on a 7890B chromatography system (Agilent Technologies, Santa Clara, CA, USA) interfaced to a time-of-flight mass spectrometer Pegasus BT 4D (LECO, USA), which was tuned according to the manufacturer’s recommendation using perfluorotributylamine (PFTBA). Automated injections are performed with an L-PAL3 autosampler (CTC Analytics AG, Zwingen, Switzerland) with 10 µL syringe (model 701 FN CTC SYR, Hamilton, Bonaduz, Switzerland). As a solvent prewash, we used pyridine and methanol, three washes (5 µL) before and ten washes (5 µL) after injection. Each sample (1 µL) was injected through the glass liner (Restek, Bellefonte, PA, USA) under split mode (50:1). Helium (6.0 grade) was used as a carrier gas, and its constant flow of 1 mL/min was maintained throughout the run. The oven was initially heated up to 60 °C, the equilibration time was 1 min, and the temperature ramped at the rate of 10 °C/min to the final temperature of 280 °C with a hold time of 12 min. The first-dimension column was 30 m long Restek Rxi-5Sil MS (Restek, Bellefonte, PA, USA), and the second-dimension column was 2 m long Restek Rxi-17Sil MS (Restek, Bellefonte, PA, USA). The transfer line of the time of MS was set at 280 °C, with a solvent delay of 350 s. The ion source temperature was 250 °C. After a solvent delay of 350 s, spectra were collected at 200 scans per second from 35–700 *m*/*z*. Ion source filament energy was set to 70 eV.

### 2.5. Data Processing

Obtained spectrum files were processed by ChromaTOF (v. 5.51, LECO, St. Joseph, MI, USA) for deconvolution, peak picking, alignment, and primary database searching. Only those finds were selected for which the signal-to-noise ratio exceeded 10. Identifications were made using the components of the NIST mass spectral and retention index database (mainlib, replib) and Leco-Fiehn rtx5 library [8]. Only those hits were considered reliable if their forward and reverse similarity exceeded 700. Retention indexes were used as an additional control for the reliability of identification: findings with the difference between the library and experimental retention indices exceeding 100 were excluded from consideration. Further, from the obtained list of identifications, only metabolites detected in all 18 technical repetitions of the GC×GC-MS analysis were selected. When evaluating reproducibility, we verified that chromatographic peak areas (AUCs) were calculated from the same, most intense ions in all technical repetitions. For this process, we analyzed extracted ion chromatograms (XICs) created by plotting the intensity of the signal of chosen most intensive *m*/*z* value in a series of mass spectra recorded as a function of retention time [9].

Statistical analyses (calculation of coefficients of variation) and plots (boxplot and scatter plots) were performed using the R software environment (version 4.0) to evaluate the repeatability of the methods. The coefficient of variation (CV) was calculated based on three technical repetitions of GC×GC-MS analysis. CVs (%) were calculated using the standard formula: CV = (Standard Deviation/Mean) × 100. The results were visualized using box-and-whiskers and scatter plots. To normalize AUCs, we calculated the following: NormAUC_i_ = (AUC_i_ − MIN(AUC))/(MAX(AUC) − MAX(AUC)), where AUC = (AUC_1_,...,AUC_31_), MIN(AUC) and MAX(AUC) are minimum and maximum values of AUC, correspondingly, and NormAUC_i_ is our ith normalized data. This formula brings the data to the range [0;1]. Statistical analyses and plots were performed using the R software environment (version 4.0) [10].

## 3. Results and Discussion

Two-dimensional chromatograms of low molecular weight plasma components obtained by GC×GC-MS contain several thousand peaks, but most remain unannotated [11]. At the stage of data processing, when searching against libraries, it is possible to routinely annotate ca. 200 biologically significant metabolites [12]. Of them, 31 TMS derivatives of 29 core blood plasma metabolites that meet strict reliability requirements (manual curation of identification candidates and control of retention indexes) were selected. A complete list of monitored compounds is presented in Table 1.

Table 1 lists the methoximation and silylation status for each metabolite, the HMDB ID according to the human metabolome database, and the subclass according to the ClassyFire system. In addition, GC×GC-MS parameters (retention times for both chromatographic dimensions and actual masses selected by the ChromaTOF software) of TMS derivatives are also presented. Characteristic *m*/*z*-values were used to calculate AUCs. For most identifications, the typical for all TMS-derivative ions (*m*/*z* = 73) turned out to be the most intense. This ion is routinely used as a quantitative characteristic in assessing the content of metabolites along with more specific ions [13,14].

According to the ClassyFire system, the list of monitored metabolites includes 13 amino acids, seven carbohydrates, two alpha amino acids, and one representative of dicarboxylic acids, beta hydroxy acids (as well as medium chain beta hydroxy acids), fatty acids, indolyl carboxylic acids, alcohols, and ureas. The amino acid tryptophan is presented as two stable silyl derivatives (2TMS and 3TMS). There are also two signals for glucose, which, based on the literature data, refer to two stereoisomers [7].

**Figure 2:**
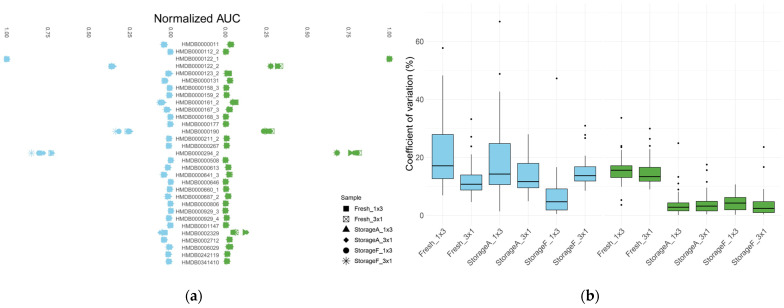
The consideration of various options for injecting a sample into the system: (**a**) normalized areas under the curves (AUCs) of chromatographic peaks of metabolites under study; (**b**) locality and spread of coefficient of variations calculated for metabolite signals. We compared the abundance of metabolites’ derivatives immediately after sample preparation (boxes with the “Fresh” prefix) and after a day of storage (boxes with the “StorageA” and “StorageF” prefixes in the case of storage at room temperature and in the refrigerator, respectively). Different injection schemes were also examined: three subsequent injections from the same vial or each technical repetition obtained from an intact vial, “3 × 1” and “1 × 3” postfixes, respectively. The blue boxes refer to the samples, during the injection of which the syringe was washed with methanol and the green ones—with pyridine. The black dots on the graph represent outliers.

For this core of metabolites, we compared the areas under the curves (AUCs) of chromatographic peaks (Figure 2a) and their reproducibility in technical repetitions (Figure 2b) to evaluate the influence of storage and injection conditions on the results obtained. The values of CVs for each metabolite obtained under different experimental conditions are presented in Supplementary Table S1.

Figure 2a shows the areas under the chromatographic curves for selected metabolites (averaged over three technical replicates and normalized). Without internal standards, it is impossible to quantify the concentrations of these metabolites reliably; however, it is clear that AUCs of TMS derivatives of glucose and urea are significantly higher than those of other molecules, which is in good agreement with the literature data [12,15].

### 3.1. Effect of Storage

The stability of TMS derivatives is an important parameter that determines whether sample preparations can wait hours in a queue before injection. We compared freshly prepared samples and samples that were stored for 24 h (at ambient temperature and in the freezer, +4 °C). We did not notice a significant loss of peak areas when injecting freshly prepared and stored samples (the average difference in AUC was 10%) in each type of experiment. Let us turn to the reproducibility of the signal, then in the case of storage of samples. Regardless of the temperature conditions, minor variability is observed during washing with pyridine (average CV values between those repeats were 4–4.5%), while in the case of freshly prepared samples, it was 15–16%. When using methanol as a flushing liquid, no patterns were observed between time and storage conditions prior to the analysis of the samples—the average CV was 15%. However, it can be unequivocally stated that the reproducibility improves significantly for L-Arabinose if the sample is preliminarily stored at +4 °C (from 27–40% to 7%).

### 3.2. Effect of Solvent

The second criterion was syringe wash: we tested methanol and pyridine as popular solvents. When the samples were analyzed immediately after preparation, no significant differences between pyridine and methanol were observed in most metabolites (average CVs were 15.5% and 16.8%, respectively). The increased difference between repetitions of analysis of freshly prepared samples is likely due to the continuation of the reaction, which requires more time. In samples stored for 24 h at ambient temperature and at +4 °C, there is a noticeable decrease in the coefficient of variation for the absolute majority of metabolites when washing the system with pyridine (the average CV was 4.3% vs. 14% when using methanol). On average, the AUCs in the samples where pyridine was used as a washing solvent were 60% lower than when the syringe was washed with methanol.

### 3.3. Effect of Vial

The contribution of the type of sample injection into the chromatographic system is noticeable in samples that were analyzed with methanol as the washing liquid. Both in the case of keeping the samples ambient for 24 h and in the case of analysis immediately after preparation, when injected from an intact vial, the CVs of metabolites were noticeably lower than after three sequential injections from the same vial (12.8% and 21%, respectively, in freshly prepared samples, 13.9%, and 19.6 %, respectively, in samples kept ambient). When using pyridine as a washing solvent, no such trends were observed, CVs between a three-time injection from the same vial and single injections from three intact vials were comparable (15.1% and 15.9%, in freshly prepared samples, 4.3%, and 4.4%, in samples stored at room temperature). Thus, when using pyridine as a wash solvent, it is possible to inject three times from one vial without significant consequences for reproducibility.

## 4. Conclusions

Two-dimensional gas chromatography coupled with mass spectrometry (GC×GC/TOF-MS) has proven to be one of the reliable and robust techniques for performing metabolomic analysis [2] due to the enhanced separation power of two dimensions chromatography and fast mass spectrometry acquisition. Due to its robustness, the GCxGC-MS approach is becoming more widely used to identify and quantify metabolites. The metabolomics community is on the verge of introducing high-performance omics methods into clinical practice. Today, it is especially valuable to evaluate the influence of various laboratory factors on the sensitivity, accuracy, and reproducibility of compound-specific quantification of small molecules by GC-MS.

As far as the authors know, this is the first study conducted to investigate the optimal conditions for storing and injecting prepared samples: this part of the experiment is deprived of the attention of researchers compared to the storage of the sample itself and its sample preparation. We have analyzed fluctuations in chromato–mass spectrometric peaks of various small molecules routinely analyzed in metabolomic studies, which occur during prolonged storage of the sample, and take into account the details of sample injection into the analytical system.

As a result of our study, we decided that the overall picture of the analysis of samples will be less variable if they are “exposed” in time and when pyridine is collected as a washing solvent. Storage, in this case, may not be favorable for the qualitative analysis of several metabolites, e.g., Glycine and L-Tryptophan, for which AUCs were decreased by 20–25%. At the same time, it should be noted that using pyridine as a washing solvent storage in the cold also may result in ca. 5% decrease in AUCs. It is also worth noting that triple injection from the same vial does not increase CV when using pyridine as a solvent, which makes it possible to simplify the sample preparation procedure without negative consequences on the results. Using methanol as a washing syringe solvent gives more scattered results than pyridine. When choosing methanol, technical repetitions should be performed with sampling from separate, intact vials. Thus, on the example of the core of blood plasma metabolites representing various classes of chemical substances, we have formed general recommendations for setting up experiments on metabolomic profiling when it is necessary to analyze a different number of samples, where questions of their optimal storage and delayed start certainly arise.

## Figures and Tables

**Table 1:** GC×GC-MS identifications of selected blood plasma metabolites.

Compound	Modifications	HMDB ID	Subclass	Retention Time, s		Actual Masses (XIC), *m*/*z*
				1D	2D	
3-Hydroxybutyric acid	2TMS	HMDB0000011	Beta hydroxy acids and derivatives	497.99	1.95	73
4-Aminobutanoic acid	2TMS	HMDB0000112	Amino acids, peptides, and analogues	509.99	1.91	130
D-Glucose	MOX. 5TMS	HMDB0000122	Carbohydrates and carbohydrate conjugates	1053.95	1.79	73
D-Glucose	MOX, 5TMS	HMDB0000122	Carbohydrates and carbohydrate conjugates	1065.95	1.80	73
Glycine	2TMS	HMDB0000123	Amino acids, peptides, and analogues	461.99	1.99	102
Glycerol	3TMS	HMDB0000131	Carbohydrates and carbohydrate conjugates	601.98	1.79	73
L-Tyrosine	3TMS	HMDB0000158	Amino acids, peptides, and analogues	1069.95	2.19	73
L-Phenylalanine	2TMS	HMDB0000159	Amino acids, peptides, and analogues	869.97	2.30	73
L-Alanine	2TMS	HMDB0000161	Amino acids, peptides, and analogues	449.99	1.89	116
L-Threonine	3TMS	HMDB0000167	Amino acids, peptides, and analogues	693.98	1.87	73
L-Asparagine	3TMS	HMDB0000168	Amino acids, peptides, and analogues	901.97	2.18	73
L-Histidine	3TMS	HMDB0000177	Amino acids, peptides, and analogues	1061.95	2.46	73
Lactic acid	2TMS	HMDB0000190	Alpha hydroxy acids and derivatives	410.00	1.90	73
Inositol	6TMS	HMDB0000211	Alcohols and polyols	1165.95	1.78	73
Pidolic acid	2TMS	HMDB0000267	Amino acids, peptides, and analogues	793.97	2.57	73
Urea	2TMS	HMDB0000294	Ureas	561.99	2.43	171
Ribitol	5TMS	HMDB0000508	Carbohydrates and carbohydrate conjugates	941.96	1.71	73
Erythronic acid	TMS	HMDB0000613	Carbohydrates and carbohydrate conjugates	825.97	1.84	73
L-Glutamine	3TMS	HMDB0000641	Amino acids, peptides, and analogues	965.96	2.22	73
β-L-Arabinose	MOX, 4TMS	HMDB0000646	Carbohydrates and carbohydrate conjugates	901.97	1.80	73
D-Fructose	MOX, 5TMS	HMDB0000660	Carbohydrates and carbohydrate conjugates	1037.96	1.78	73
L-Leucine	2TMS	HMDB0000687	Amino acids, peptides, and analogues	597.98	1.90	158
Tetradecanoic acid	TMS	HMDB0000806	Fatty acids and conjugates	1005.96	2.15	73
L-Tryptophan	2TMS	HMDB0000929	Indolyl carboxylic acids and derivatives	1221.94	3.14	73
L-Tryptophan	3TMS	HMDB0000929	Indolyl carboxylic acids and derivatives	1229.94	2.57	73
Aminomalonic acid	MOX, TMS	HMDB0001147	Amino acids, peptides, and analogues	757.97	2.11	73
Oxalic acid	2TMS	HMDB0002329	Dicarboxylic acids and derivatives	473.99	2.21	73
1,5-Anhydrosorbitol	4TMS	HMDB0002712	Carbohydrates and carbohydrate conjugates	1021.96	1.95	73
N2-Acetyl-L-glutamine	4TMS	HMDB0006029	Amino acids, peptides, and analogues	941.96	1.84	73
D-Mannonic acid	4TMS	HMDB0242119	Medium-chain hydroxy acids and derivatives	1085.95	1.99	73
2-Hydroxybutanoic acid	2TMS	HMDB0341410	Alpha hydroxy acids and derivatives	469.99	1.94	73

## Data Availability

The data supporting this study’s findings are available from the corresponding author upon request or via Mendeley Data links (https://data.mendeley.com/datasets/4y8b27v7mg/draft?a=6df2e93a-e6b5-4ff0-9a80-747a42f81b62—pyridine dataset; https://data.mendeley.com/datasets/thrhrdt8kn/draft?a=e01da1fa-7d6c-4e63-9f5d-6077371f4507—methanol dataset, both accessed on 5 December 2022).

## Supplementary Materials

The following supporting information can be downloaded at: https://www.mdpi.com/article/10.3390/metabo13010075/s1; Table S1: Coefficients of variation of TMS derivatives of selected metabolites.
